# Supplementary material for: Chronic kidney disease among HIV-positive Zambian adults with tenofovir-associated nephrotoxicity at University Teaching Hospital (UTH) in Lusaka
Source: PLoS One. 2025 Aug 14;20(8):e0330356. doi: 10.1371/journal.pone.0330356 (PMC12352757; doi:10.1371/journal.pone.0330356)
Supplement: Attachment — Submitted filename: Response to Reviewers.docx [file pone.0330356.s003.docx]

**Rebuttal Letter: Response to Reviewers**

**Manuscript Title:** *Chronic Kidney Disease among HIV-positive Zambian adults with Tenofovir-associated Nephrotoxicity at University Teaching Hospital (UTH) in Lusaka*

**Manuscript Number:** PONE-D-25-19997

**Corresponding Author:** Edgar Muchinta

**Date:** 21/07/2025

Dear Dr. Abdulwasiu Bolaji Tiamiyu and Reviewers,

We thank you for the thoughtful evaluation of our manuscript and the opportunity to revise and resubmit to PLOS ONE. Below, we provide a detailed, point-by-point response to the comments raised by the reviewers and address all editorial requirements. Changes have been made to the manuscript as indicated, and a marked-up version with tracked changes is included alongside a clean copy.

**Reviewer #1 Comments and Author Responses**

**1. Concern:** Use of external comparison with Hepatitis B patients for CKD incidence rates.

**Response:** We appreciate this observation. The comparison with Hepatitis B patients was derived from a previously published study and served only as contextual framing for our incidence rate in Table 2. We have now clarified this in the revised **Discussion** section. Furthermore, the **Limitations** section now acknowledges the difference in pathophysiology and treatment exposures between Hepatitis B and HIV populations and notes that future studies should incorporate HIV-specific control cohorts.

**2. Concern:** Missing clinicodemographic characteristics such as CD4 count, viral load, smoking status, alcohol intake, proteinuria, and comorbidities.

**Response:** Thank you for this valuable recommendation. We have addressed this by:

- Explicitly stating in the revised **Methodology** that hypertension, diabetes mellitus, and other chronic comorbidities were part of our exclusion criteria, and this step was taken to reduce confounding.
- Expanding the **Limitations** section to explain that CD4 count, viral load, proteinuria, smoking status, alcohol use, herbal medication intake, and haematocrit were not consistently recorded due to the retrospective nature of the study and therefore excluded from analysis. We noted that future prospective studies could overcome this gap to enable deeper univariate analyses.

**Reviewer #2 Comments and Author Response**

We thank Reviewer #2 for their encouraging feedback and endorsement of our study’s technical rigor, statistical methodology, and scholarly communication. We were pleased to see that the data were deemed appropriately analyzed and the manuscript clearly written and methodologically sound.

**Editorial Requirements Response**

**1. Abstract Consistency:** We have ensured that the abstract in the manuscript and the online submission form are now fully aligned.

**2. Competing Interests Statement:** We updated this section to: *“The authors have declared that no competing interests exist.”*

**3. Data Availability Statement:** We included the statement: *“All data are in the manuscript and/or supporting information files.”* placed under a dedicated **Data Availability** heading.

**4. Formatting Compliance:** We reviewed and updated the manuscript to comply with PLOS ONE formatting guidelines, including file naming conventions and reference layout.

**5. Figure Standards:** All figure files have been verified to comply with the journal's figure submission criteria using PACE.

We trust these revisions fully address the concerns raised and strengthen the manuscript for publication. We remain grateful to the reviewers for their constructive comments and to the editorial team for their guidance and professionalism.

Please feel free to contact us if further clarification is required.

Kind regards, **Edgar Muchinta** on behalf of all co-authors Email: emuchinta@gmail.com
